# Pneumonia in severely injured patients with thoracic trauma: results of a retrospective observational multi-centre study

**DOI:** 10.1186/s13049-019-0608-4

**Published:** 2019-03-14

**Authors:** Sebastian Wutzler, Felix M. Bläsius, Philipp Störmann, Thomas Lustenberger, Michael Frink, Marc Maegele, Matthias Weuster, Jörg Bayer, Michael Caspers, Andreas Seekamp, Ingo Marzi, Hagen Andruszkow, Frank Hildebrand

**Affiliations:** 1 Department of Trauma, Hand and Reconstructive Surgery, Hospital of the Johann Wolfgang Goethe-University, Theodor-Stern-Kai 7, D-60590 Frankfurt, Germany; 2 Department of Trauma and Reconstructive Surgery, University Hospital RWTH Aachen, Pauwelsstraße 30, D-52074 Aachen, Germany; 3 Department of Trauma, Hand and Reconstructive Surgery, University Hospital Marburg, Baldingerstraße, D-35043 Marburg, Germany; 4 Department of Trauma and Orthopaedic Surgery, Cologne-Merheim, Medical Centre (CMMC), Ostmerheimer Str. 200, D-51109 Köln, Germany; 5 Department of Trauma Surgery, University Hospital Schleswig-Holstein, Campus Kiel, 24105 Kiel, Germany; 6 Department of Orthopaedics and Trauma Surgery, Medical Centre Albert-Ludwings-University of Freiburg, Sir-Hans-A.-Krebs-Straße, D-79106 Freiburg, Germany

**Keywords:** Pneumonia, Thoracic trauma, ISS, AIS, ICU

## Abstract

**Background:**

While the incidence and aspects of pneumonia in ICU patients has been extensively discussed in the literature, studies on the occurrence of pneumonia in severely injured patients are rare. The aim of the present study is to elucidate factors associated with the occurrence of pneumonia in severely injured patients with thoracic trauma.

**Setting:**

Level-I University Trauma Centres associated with the TraumaRegister DGU®.

**Methods:**

A total of 1162 severely injured adult patients with thoracic trauma documented in the TraumaRegister DGU® (TR-DGU) were included in this study. Demographic data, injury severity, duration of mechanical ventilation (MV), duration of ICU stay, occurrence of pneumonia, bronchoalveolar lavage, aspiration, pathogen details, and incidences of mortality were evaluated. Statistical evaluation was performed using SPSS (Version 25.0, SPSS, Inc.) software.

**Results:**

The overall incidence of pneumonia was 27.5%. Compared to patients without pneumonia, patients with pneumonia had sustained more severe injuries (mean ISS: 32.6 vs. 25.4), were older (mean age: 51.3 vs. 47.5) and spent longer periods under MV (mean: 368.9 h vs. 114.9 h). Age, sex (male), aspiration, and duration of MV were all independent predictors for pneumonia occurrence in a multivariate analysis. The cut-off point for duration of MV that best discriminated between patients who would and would not develop pneumonia during their hospital stay was 102 h. The extent of thoracic trauma (AIS_thorax_), ISS, and presence of pulmonary comorbidities did not show significant associations to pneumonia incidence in our multivariate analysis. No significant difference in mortality between patients with and without pneumonia was observed.

**Conclusions:**

Likelihood of pneumonia increases with age, aspiration, and duration of MV. These parameters were not found to be associated with differences in outcomes between patients with and without pneumonia. Future studies should focus on independent parameters to more clearly identify severely injured subgroups with a high risk of developing pneumonia.

**Level of evidence:**

Level II - Retrospective medical record review.

## Background

Of multiple trauma patients admitted to hospitals, 44.8% suffer from an associated thoracic injury. Thoracic trauma is the second most common injury in severely injured patients in Germany [1]. Extent of thoracic injury is subject to considerable individual variation and is characterized by impairment of lung function, pleural effusion, inadequate blood oxygenation, increased release of inflammatory cytokines, and increased inflammatory cell recruitment [2–4]. In addition to injury of the osseous thorax, thoracic organs may also be affected. Thoracic trauma has been associated with longer durations of mechanical ventilation (MV) and respiratory complications such as pneumonia or acute respiratory distress syndrome (ARDS). These complications correlate to mortality rates that can be as high as 24% [5–7]. The incidence of ventilator-associated pneumonia (VAP), a frequent nosocomial infection, was shown to be highest in trauma patients, and represented a predisposing factor for the development of trauma-associated ARDS and multiple organ failure (MOF) [8–10]. The early identification of high-risk patients as well as influenceable factors that predispose patients toward developing pneumonia is of significant importance for post-traumatic pneumonia risk reduction. The heterogeneity of trauma patients (e.g. injury severity and distribution, age, pre-existing disease), however, poses a considerable challenge to the identification of discrete risk factors.

Comprehensive meta-analyses on the incidence, clinical course, and outcomes in specific subgroups of intensive care unit (ICU) patients with pneumonia are rare; most reports base their analyses on heterogeneous patient groups or include just a small number [11–13] of cases. Published data on pneumonia incidence in trauma patients offers statistics anywhere from 8% to more than 50% [14–23]. Few studies focus on severely injured patients with significant thoracic trauma, despite the fact that chest injuries have been intensively reviewed as predisposing factors for pulmonary infection [24–28]. Data on the incidence, independent risk factors, and outcomes of respiratory complications (e.g. pneumonia) in a large cohort of these patients is of urgent importance to define benchmarks by which clinical data can be compared, apply to collective quality management, and set reference data on the efficiency of new therapies such as prophylactic positive end-expiratory pressure (PEEP) ventilation, prone positioning, kinetic therapy, and the use of antibiotics.

In the present study, we collected epidemiological, clinical, and outcome data from a large group of severely injured ICU patients with thoracic trauma to determine the rate of respiratory complications therein in a retrospective, multi-centre approach. We focused on pneumonia rates, risk factors, and underlying pathogens in bronchoalveolar lavage fluid (BALF).

## Methods

This study was produced by the Trauma Committee of the German Interdisciplinary Association for Intensive and Emergency Medicine (Deutsche Interdisziplinäre Vereinigung für Intensiv- und Notfallmedizin, DIVI). In December 2015, the committee embarked on a retrospective, observational study of the quality of care in patients with thoracic trauma that underwent MV from the years 2010 to 2014. Six German hospitals (Aachen, Cologne, Frankfurt, Freiburg, Kiel and Marburg) contributed patient data for analysis. All participating hospitals were Level I trauma centres.

This study follows the guidelines of the revised UN declaration of Helsinki in 1975 and its latest amendment in 2013 (64th general meeting). The following approvals were provided by each institution’s ethical committee: Aachen: EK 346/15, Cologne 18/2016, Frankfurt: 220/16, Kiel: B 248/16, Freiburg: 275/16, Marburg: No ethics committee vote necessary for retrospective chart analysis. Due to the study’s retrospective nature, informed consent from the study participants was not required, in accordance with the ethical approvals from the institutional committees.

All six hospitals participate in the nationwide TraumaRegister DGU® (TR-DGU) of the German Trauma Society (Deutsche Gesellschaft für Unfallchirurgie, DGU). The TR-DGU was founded in 1993 with the aim of creating a multi-centre database for pseudonymized and standardized documentation of severely injured patients for the purposes of research. Participating hospitals are primarily located in Germany (90%), but a rising number of hospitals from other countries have begun to contribute data as well, to include Austria, Belgium, China, Finland, Luxembourg, Slovenia, Switzerland, The Netherlands, and the United Arab Emirates. Currently, approximately 30,000 cases from more than 900 hospitals are entered into the database per year. Participation in the TR-DGU is voluntary; however, hospitals associated with the TraumaNetzwerk DGU® are obligated to enter at least one basic dataset for quality assurance purposes.

Data were collected prospectively over four consecutive time phases from site of injury until discharge from hospital, as follows: A) pre-hospital phase, B) emergency room and initial surgery, C) intensive care unit, and D) discharge. Documentation included detailed information on demographics, injury pattern, comorbidities, pre- and in-hospital management, course of care while in the intensive care unit, relevant laboratory findings (including data on transfusion), and clinical outcome for each individual. Inclusion criteria were: admission to hospital via emergency room with subsequent ICU/ICM care, or entrance to the hospital with vital signs and death prior to admission to the ICU.

Infrastructure for documentation and data management was provided by the Academy for Trauma Surgery (AUC – Akademie der Unfallchirurgie GmbH), a company affiliated with the German Trauma Society. Scientific leadership draws from the Committee on Emergency Medicine, Intensive Care, and Trauma Management (NIS Committee) of the German Trauma Society. Participating hospitals submitted pseudonymized data into a central database via web-based application. Scientific data analysis was approved according to a peer review procedure established by the NIS Committee.

While the TR-DGU has been used as database for numerous analyses and publications on severely injured patients [29], analyses are restricted to what has been uploaded by the institution, and documentation with regards to respiratory clinical course and complications is limited. For the purposes of this study, each individual hospital retrieved its own data from the database and added relevant information from clinical documentation systems. The study group chose to retroactively include all patients from the six study sites that fulfilled the following criteria:

- Age ≥ 18 years
- Primary admissions
- Abbreviated Injury Scale (AIS_thorax_) ≥ 3
- Admitted between the years of 2010 and 2014
- ICU treatment ≥1 day

For these patients, data was retrieved from the TR-DGU by the hospitals involved in this study following approval from the participating hospitals and the TR-DGU. Data were complemented by a review of the patients’ records from each hospital. Additional data were pseudonymized and added to the TR-DGU data as follows:

- Bronchoalveolar lavage (BAL) yes/no
- BAL positive yes/no, if yes, pathogen details
- Aspiration yes/no
- Tracheotomy yes/no
- MV in hours
- Re-intubation for respiratory needs yes/no
- Pneumonia yes/no (CPIS > 6 or as described below), if yes: VAP/hospital acquired pneumonia (HAP)/other, and early vs. late onset
- CPIS (maximum)
- Discharge on MV yes/no
- Pulmonary comorbidities (predefined list), if yes: n
- Worst Horowitz Index value
- Highest PEEP
- In case of discharge on MV, worst Horovitz Index and highest PEEP as determined from the first 14 days of MV

Overall injury severity was calculated by the Injury Severity Score (ISS) as described by Baker et al. [30]. The Clinical Pulmonary Infection Score (CPIS) was used to assess the occurrence of pneumonia in ventilated patients [31]:

- Body temperature (°C): 36.5–38.4 = 0 point; 38.5–38.9 = 1 point; > 39.0 or ≤ 2 points
- White blood cell count (microscopy): 4000-11,000/mm^3^ = 0 point; < 4000/mm^3^ or > 11,000/mm^3^ = 1 point; either < 4000 or > 11,000/mm^3^ plus band forms ≥50% = 2 points
- Tracheal Secretions: None or scant = 0 point; tracheal secretion with less purulence = 1 point; abundant purulent secretion = 2 points.
- PaO_2_/FiO_2_: > 240, ARDS or pulmonary contusion = 0 point; ≤240 and no ARDS = 2 points
- Chest Radiograph: No infiltrate = 0 point; diffuse (or patchy) infiltrate = 1 point; localized infiltrate = 1 point
- Progession in pulmonary infiltration: no = 0 point; yes (after the exclusion of HF and ARDS) = 2 points
- Pathogenic bacteria in tracheal aspirate culture: no or few pathogenic bacteria = 0 point; moderate or high levels of pathogenic bacteria = 1 point; pathogenic bacteria to be seen in Gram staining = add 1 point

A total of > 6 pts. was accepted as pneumonia.

In non-ventilated patients, pneumonia was defined as the presence of new progressive infiltrate accompanied by at least two of the following symptoms:

- Purulent respiratory secretions
- Body temperature ≥ 38 °C or ≤ 35 °C
- Leucocytosis (white blood cell count of ≥10,000/mm^3^) or leucopoenia (white blood cell count of ≤4500/mm^3^, or more than 15% immature neutrophils)

Standard for numeric results of BAL was utilized following recommendations of the German Society of Hygiene and Microbiology [32].

Continuous variables were compared using Student’s *t*-test and categorical variables analysed using the Chi-square test. Results are presented as mean plus or minus standard deviation (SD) with a confidence interval (CI) of 95%. Analyses were performed with SPSS (Version 25.0, SPSS, Inc.) for Windows. A two-tailed *p*-value ≤0.05 was considered significant. The interpretation of results, however, should focus on clinically relevant differences rather than on significant *p*-values.

## Results

In total, 1162 patients from the six Level I trauma centres met the inclusion criteria during the 5 year observation period. Of these patients, 1119 (96.3%) presented with complete pneumonia occurrence datasets; these datasets were entered for analysis. Basic epidemiological and outcome data were analysed for the whole cohort. The majority of patients were male (76.2%) and suffered from thoracic trauma; mean age was 48.4 years (mean: 48.39, range: 18 to 94, SD ± 18.65). Basic demographic data are detailed in Table 1.

**Table 1:** Basic data of study collective, means given with SD and 95% CI, ^a^complete data on pneumonia (96.3%), ^b^median with 25th 75th percentile

Basic data	
n	1162 (1119^a^)
ISS (pts.)	27.5 ± 12.6
RISC II mortality prognosis (%)	11.6
Hospital mortality (%)	8.9 (7.2–10.5)
SMR	0.764 (0.623–0.905)
AIS_thorax_ (pts.)	3.6 ± 0.8
Male (%)	76.2 (73.8–78.7)
Age (yrs.)	48.4 ± 18.7
Penetrating trauma (%)	3.6 (2.5–4.6)
ICU (days)	10.5 ± 12.7
GOS^b^	4 (4–5)

About one quarter of patients (308/1119, 27.5% [95% Cl, 24.9–30.1%]) fulfilled the criteria for a pneumonia diagnosis. Classification of the type of pneumonia was possible in 290 patients. In 24.5% of these cases, HAP (while not ventilated) was diagnosed, 42.1% were early-onset VAP (1–4 days of MV), and 33.4% were late-onset VAP (5 days or more). Table 2 compares epidemiological, clinical, and outcome data from patients with pneumonia versus patients without pneumonia. Patients with pneumonia were significantly more severely injured (mean ISS: 32.6 ± 12.8 vs. 25.4 ± 11.8, *p* <  0.001) and older (mean age in years: 51.3 ± 18.9 vs. 47.5 ± 8.5, *p* = 0.002). Length of ICU stay (mean in days: 21.8 ± 15.4 vs. 6.3 ± 8.1, *p* <  0.001) and duration of MV (mean in hours: 368.9 ± 303.1 vs. 114.9 ± 161.4, *p* <  0.001) were significantly longer in the pneumonia group. Incidences of reintubation and tracheotomy were significantly higher in patients with pneumonia. In contrast, hospital mortality was only slightly higher in the pneumonia group (mean: 10.1%, 6.7 to 13.4 vs. 7.9%, 6.0 to 9.7, *p* = 0.3).

**Table 2:** Comparison of patients with and without pneumonia, means given with SD and 95% CI, ^a^median with 25th – 75th percentile

	No pneumonia	Pneumonia	*p* value
N	811 (72.5%)	308 (27.5%)	–
ISS (pts.)	25.4 ± 11.8	32.6 ± 12.8	< 0.001
RISC II mortality prognosis (%)	8.8	19.5%	< 0.001
Hospital mortality (%)	7.9% (6.0–9.7)	10.1% (6.7–13.4)	0.30
AIS_head/neck_ (pts.)	1.4 ± 1.7	2.3 ± 1.9	< 0.001
AIS_thorax_ (pts.)	3.5 ± 0.7	3.7 ± 0.8	< 0.001
AIS_abdomen_ (pts.)	0.9 ± 1.4	1.0 ± 1.4	0.88
AIS_extremities_ (pts.)	1.6 ± 1.4	2.0 ± 1.4	< 0.001
Male (%)	74.4 (71.3–77.4)	80.8 (76.4–85.2)	0.028
Age (yrs.)	47.5 ± 18.5	51.3 ± 18.9	0.002
Penetrating trauma (%)	4.3 (2.9–5.6)	1.3 (0.0–2.6)	0.027
LOS (days)	16.3 ± 15.1	29.1 ± 19.4	< 0.001
ICU (days)	6.3 ± 8.1	21.8 ± 15.4	< 0.001
Time on ventilation (h, all)	58.0 ± 128.2	346.0 ± 306.7	< 0.001
Time on ventilation (h, ventilated patients)	114.9 ± 161.4	368.9 ± 303.1	< 0.001
GOS^a^	5 (4–5)	4 (3–5)	< 0.001
BAL (%)	4.9 (3.4–6.4)	79.5 (75.0–84.1)	< 0.001
Aspiration (%)	3.3 (2.1–4.6)	21.1 (16.5–25.7)	< 0.001
Tracheotomy (%)	4.6 (3.1–6.0)	44.8 (39.3–50.4)	< 0.001
Reintubation (%)	2.5 (1.4–3.5)	17.9 (13.6–22.1)	< 0.001
Discharge on ventilation (%)	2.2 (1.2–3.2)	17.3 (13.0–21.5)	< 0.001
Worst Horovitz Index	282.9 ± 135.7	137.8 ± 83.4	< 0.001
ECMO (%)	0.4 (0.0–0.8, *n* = 3)	0.0 (*n* = 0)	1.0

In the majority (79.5%) of patients with pneumonia (73.1% of VAP patients), a BAL for microbiological culture was performed. In 39 of these cases, microbiological cultures were negative. In the remaining 206 cases (84.1, 95% Cl 79.5–88.7%), cultures were positive for at least one pathogen (Table 3), the most common single species of which was *Staphylococcus aureus* (18.1%), followed by various gram-negative bacteria. In 10.8% of these cases, *Candida* species were identified.

**Table 3:** Isolated Pathogens in Bronchoalveolar Lavages

Name	n (%)	Type
Enterobacter species	121 (36.4)	Gram-negative
*Escherichia coli*	46 (13.9)	
*Klebsiella pneumonia*	38 (11.5)	
Serratia species	14 (4.2)	
*Staphylococcus aureus*	60 (18.1)	Gram-positive
Candida species	36 (10.8)	Fungus
*Haemophilus influenza*	29 (8.7)	Gram-negative
Pseudomonas species	22 (6.6)	Gram-negative

Isolated pathogens in qualitative bronchoalveolar lavages from patients developing pneumonia during intensive care treatment; pathogens wit *n* < 10: *Streptococcus, Enterococcus, Neisseria, Aspergillus, Anaerobic, Citrobacter, Proteus, Herpes, Acinetobacter, Morganella, Pneumococcus, Havnia*

**Fig. 1:**
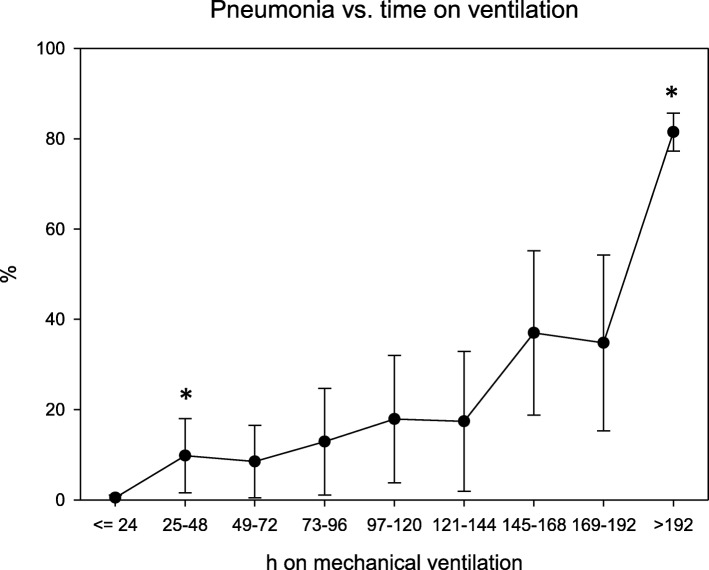
Rates of pneumonia (%) versus time on mechanical ventilation (h), 95% CI are shown, p overall < 0.001; * = significant increase to left datapoint

Duration of MV was significantly correlated to pneumonia occurrence (Fig. 1). The MV duration cut-off point that most accurately discriminated between patients who would and those who would not develop pneumonia during their hospital stay was 102 h.

**Fig. 2:**
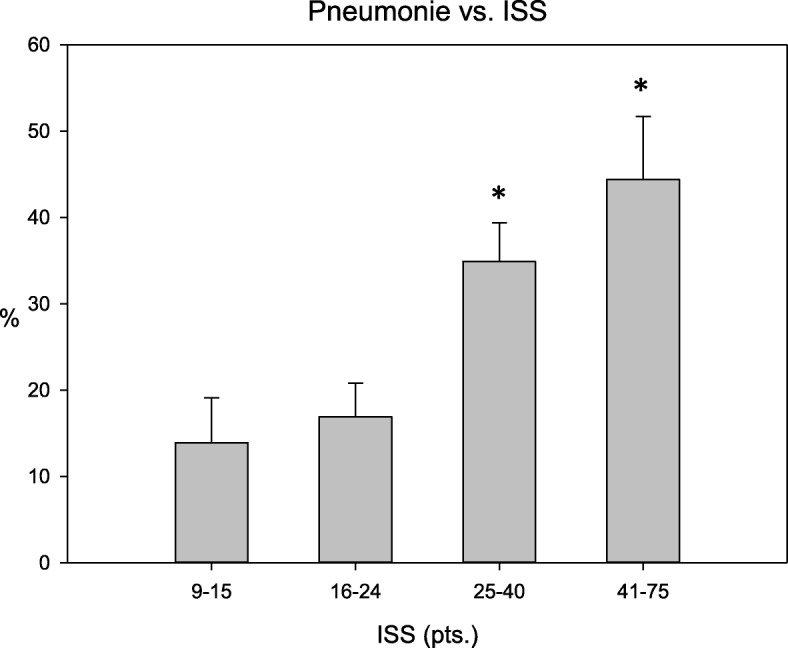
Rates of pneumonia (%) versus Injury Severity Score (pts.) in trauma patients with chest injury (AISThorax 3), 95% CI are shown, p overall < 0.001; * = significant increase to left datapoint

Overall injury severity as indicated by the ISS also significantly correlated to pneumonia incidence. Significantly higher rates of pneumonia (*n* = 178) (95% CI 31.1–51.7%, *p* <  0.001, r = 0.267) were observed in patients with an ISS of at least 25 (Fig. 2).

Multivariate logistic regression analysis with pneumonia as the dichotomous dependent variable revealed that certain independent factors were significantly associated with development of pneumonia; these were sex, age, hours of MV, and aspiration event. Severity of thoracic trauma (AIS_thorax_), ISS, and presence of pulmonary comorbidities did not demonstrate a significant association with pneumonia occurrence (Table 4).

**Table 4:** Odds ratios for parameters that showed independent association with the occurrence of pneumonia in multivariate analysis; parameters without significant association were: ISS, AIS_thorax_ = 4, AIS_thorax_ = 5, blunt/penetrating trauma mechanism, pulmonary comorbidities

Variable	Odds ratio	95% CI	*p* value
Aspiration	5.974	3.420–10.436	< 0.001
Age (per year)	1.012	1.003–1.021	0.012
Mechanical ventilation (per h)	1.005	1.005–1.006	< 0.001
Female sex	0.549	0.359–0.842	< 0.001

## Discussion

This study analysed observational data on severely injured patients with thoracic trauma collected from six German Level I trauma centres. Only a handful of previous studies, each with small samples, have investigated the incidence of pneumonia in severely injured patients. With this study, we aimed to explore the incidence of pneumonia in the largest cohort of severely injured patients with thoracic trauma investigated thus far. Our main findings can be summarized as follows:

- Sex, age, duration of MV, and aspiration were all independent predictors for occurrence of pneumonia
- ISS, ICU stay, and reintubation and tracheotomy were each significantly associated with pneumonia.
- Pneumonia in and of itself did not result in significantly different mortality rates.
- The cut-off point past which the duration of MV was most likely to predict pneumonia was 102 h.

Our study cohort yielded a similar distribution of basic epidemiological data, (mean age of about 48, approximately three-quarters male at 76%), as that found in the nationwide TR-DGU (mean age 51.2; 69% male) [1]. However, mean ISS was higher in our study as compared to the nationwide TR-DGU (27.5 vs. 18.4). This difference is likely due to the AIS_thorax_ ≥ 3 required for inclusion in our study, as severe thoracic trauma is known to be significantly associated with a higher ISS [33]. We consider our specific sample to be an important representative cohort for providing reference data for the occurrence of pneumonia in severely injured patients with thoracic trauma.

### Incidence of pneumonia

In our study, the overall incidence of pneumonia was 27.5%. In order to calculate the rate of VAP, we excluded hospital-acquired pneumonia (developed during non-ventilation phases). This resulted in a VAP rate of 20.8%, which is in alignment with reports indicating that VAPs represent between 60 and 80% of nosocomial pneumonias [34, 35].

Incidences of pneumonia in severely injured patients range from 8% to more than 50% in the literature [14–23]. Reports employing the specific inclusion criteria of the present study (severely injured patients with significant thoracic trauma) resulted in pneumonia rates between 13.2 and 45% [16, 36]. Many factors could possibly explain this discrepancy; First, previous studies focusing on severely injured patients with thoracic trauma only included a small number of patients (*n* = 79 and *n* = 223), necessarily limiting the predictive power of the results. Second, a lack of standard diagnostic criteria and consensus surrounding the definition of pneumonia permeates medical practice; this can result in significant variations in reported rates, making it challenging to identify of high risk patients. Third, incidence reporting differed among different institutions (e.g. trauma service vs. infection control) as well as whether a clinical strategy or a bacteriologic strategy was employed for VAP diagnosis [19, 20, 23]. Fourth, demographic and clinical differences within trauma populations are an important consideration. In this context, significant differences in ISS (e.g. < 16 vs > 35 points) can have a considerable impact on the duration of MV as well as the incidence of pneumonia itself [16, 17]. Lastly, therapeutic modalities can also affect the development and course of the pneumonia. In a single centre trial, effects of continuous lateral rotational therapy on patients undergoing post-traumatic courses of care were investigated [16]. Authors observed a reduction in MV duration and time spent in the ICU following this therapy. By the same token, they also reported a low incidence of VAP (13.2%). As rotational treatment was not routinely provided to patients examined in this and other studies [23], it could be inferred that this therapy might be associated with a lower VAP rate [20, 36]. This is an interesting finding that deserves future research.

We believe the high number of patients and combined clinical-diagnostic and bacteriologic parameters for diagnosis of pneumonia and VAP in this investigation has yielded important data points with regards to the incidence thereof in severely injured patients with thoracic trauma.

### Risk factors for occurrence of pneumonia

In our study, patients with pneumonia had a higher ISS, were older, and were more likely to be male. Pneumonia correlated to a two-fold decrease in Horowitz Index, a three-fold increase in time under MV, and a prolongation of ICU treatment and total length of stay (LOS). Patients with pulmonary infections were also more likely to have suffered an aspiration event and receive tracheotomy or reintubation. These aspects might contribute to the poorer clinical outcomes, measured by Glasgow Outcome Scale (GOS), observed in pneumonia patients in this study’s cohort. Our epidemiologic data was in agreement with previous studies that included severely injured patients (regardless of thoracic trauma) that found higher age, higher ISS, lower Glasgow Coma Scale (GCS), and more severe head and extremity trauma (based on AIS) to be predispositions for pneumonia [17, 18, 21]. In addition, a sex-related risk for post-traumatic infections in male patients has been well-established; beneficial effects of female hormones on the immune system have been suggested as a potential cause for this disparity between the sexes [37, 38]. With regards to clinical course, increased durations of MV as well as ICU stay and LOS in cases of post-traumatic pneumonia have also been reported [21, 22].

Among the factors discussed above, we identified aspiration, sex, age, and duration of MV as independent risk factors for the development of pneumonia. In summary, we found two influenceable risk factors for the occurrence of pneumonia: aspiration and duration of mechanical ventilation. The highest risk was observed for aspiration events (OR 5.97), which underscores data from other studies indicating aspiration as a common risk factor for the development of pneumonia [27, 39]. The importance of age, sex, and duration of MV as independent risk factors for pneumonia have been emphasised for trauma patients in many reports, regardless of the presence of thoracic trauma [26, 40–42].

In our multivariate analysis, we did not observe the association between ISS and pneumonia reported by a majority of previous studies [15, 21, 24, 27, 42, 43]. As other studies specified severe injuries to specific body regions (AIS_head_, AIS_chest_, AIS_extremity_) as being independently associated with the development of pneumonia, we surmise that injury location rather than overall severity is of greater diagnostic importance [21]. Several studies have reported preclinical intubation as an independent risk factor for the development of pneumonia [21, 27, 28]. Due to parameters specific to the German health system, wherein first responders include physicians, almost all patients in our study were intubated at the scene of injury prior to admission to the hospital, rendering us unable to reliably report on the time point of intubation.

We found no significant effect of pneumonia on post-traumatic mortality, although Revised Injury Severity Classification Score II (RISC II) mortality prognoses were much higher for the pneumonia group. This could be because RISC II prognosis considers uncontrolled bleeding a major cause of death, as is more likely to occur in our study cohort of severely injured patients [44]. Other studies have similarly found that pneumonia incidence did not influence the likelihood of post-traumatic mortality [10, 21, 26, 45]. A study by Fahr et al. (2017) did demonstrate a trend wherein pneumonia incidence was correlated to that of mortality. Fahr’s team contended that an increase in the number of pneumonia cases could partially explain the higher mortality rates observed in their study [22]. Sharpe et al. (2014), however, identified pneumonia as an independent risk factor for mortality only in females, whereas another study identified pneumonia as an independent predictor of mortality only in less severely injured patients (mean ISS < 16) [17, 41]. Similar results were reported by Gannon et al. (2004) for patients presenting with injuries of minor and moderate severity [40]. It could therefore be cautiously assumed that pneumonia exerts relevant effects on mortality only in specific subgroups of trauma patients, particularly those without severe injuries as the impact of injury on mortality in patients with a minor to moderate ISS is usually low. It follows that the effect of post-traumatic complications (e.g. pneumonia) on mortality might be substantially more relevant in these patients. It is also worth pointing out that the number of patients in each of these studies is too small to make any overarching statements about pneumonia and mortality vis-à-vis these trauma subgroups. Such findings should instead be a jumping-off point for potential future comparative studies focusing on mortality as a potential end point.

### Pathogens in BAL

In > 80% of the patients in our study with pneumonia, diagnostic BAL was performed as recommended by national guidelines for nosocomial pneumonia [46]. Pathogens were identified in 84.1% of all BAL procedures performed on this study’s cohort. In a previous multicentre study, “EU-VAP/CAP,” Koulenti et al. (2017) reported a similar detection rate [47]. The most common pathogens in our study were Enterobacteriaceae (36.4%) and *Staphylococcus aureus* (18.1%), similar to results from previous studies on severely injured patients with and without severe thoracic trauma [10, 43, 47, 48]. The high rate of gram-negative organisms (e.g. Enterobacteriaceae) is almost certainly associated with the high incidence of aspiration events in the development of pneumonia. The relevance of *Staphylococcus aureus*, particularly in patients with thoracic trauma, has been emphasized by Fahr et al. (2017) who found that subjects with contusions were more likely to grow methicillin-sensitive *Staphylococcus aureus* [36]. Polymicrobial infections presented in around one third of the cases (35.1%) in accordance with results from previous studies [47, 49].

When interpreting the pathogen spectrum, the importance of geographical origin of the study population cannot be overstated. In this context, a study from Qatar identified *Klebsiella pneumoniae* and *Hemophilus influenza* as the microorganisms most commonly associated with post-traumatic pneumonia. In our study, however, these pathogens played but a minor role [26].

### Strengths and limitations

While the large cohort for this study enables certain important statements about this patient group, this study has some relevant limitations. Retrospective registry studies are restricted to initially documented data; some points of interest may not be precise enough for solid scientific analyses, e.g. the exact time points of BAL procedure, or presentation versus definitive diagnosis of pneumonia. Additionally, in consequence of a multi-centre approach, some inconsistency in data collection is inevitable. Standardised scores (e.g. CPIS) and national guidelines were used to standardize data collection to the extent possible. Moreover, approximately 3.7% of all datasets were excluded due to missing data referring the occurrence of pneumonia. Although this might have influenced our results, we expect this bias to be of minor effect. As with any study of this nature, the data only supports association, rather than causation. Furthermore, the following diagnostic limitations need to be considered: A) initial chest X-ray might not have shown infiltrates apparent in the first 72 h, B) chronic changes on chest X-ray might have been misinterpreted as acute pneumonic infiltrates, C) presence of bacteria in sputum or endotracheal is known to be sensitive, but non-specific. This might have led to overtreatment and higher false positive rates.

Despite these limitations, this study presents a large cohort evaluating influenceable risk factors for the occurrence of pneumonia in severely injured patients with relevant thoracic trauma.

## Conclusions

This report is the first to characterise post-traumatic pneumonia and its impact on clinical course in a large cohort of severely injured patients with significant thoracic trauma. Identification of specific risk factors, details of clinical course, and most common triggering pathogens could help to better prevent, diagnose, and treat pulmonary infections in these patients. Further studies are needed to describe the incidence of pneumonia in specific subgroups of traumatized patients.

## Abbreviations

AIS: Abbreviated Injury Scale
ARDS: Acute Respiratory Distress Syndrome
AUC: Academy for Trauma Surgery (AUC – Akademie der Unfallchirurgie GmbH)
BAL(F): Bronchoalveolar lavage (fluid)
CPIS: Clinical Pulmonary Infection Score
DGU: German Trauma Society (Deutsche Gesellschaft für Unfallchirurgie, DGU)
DIVI: German Interdisciplinary Association for Intensive and Emergency Medicine (Deutsche Interdisziplinäre Vereinigung für Intensiv- und Notfallmedizin, DIVI)
GCS: Glasgow Coma Scale
GOS: Glasgow Outcome Scale
HAP: Hospital-acquired Pneumonia
ICU: Intensive Care Unit
ISS: Injury Severity Score
LOS: Length of Stay
MOF: Multiple Organ Failure
MV: Mechanical Ventilation
OR: Odds Ratio
PEEP: Positive End-expiratory Pressure
RISC II: Revised Injury Severity Classification Score II
SD: Standard deviation
TR-DGU: TraumaRegister DGU®
VAP: Ventilator-associated Pneumonia
